# The incidence of symptomatic infection with influenza virus in the Netherlands 2011/2012 through 2016/2017, estimated using Bayesian evidence synthesis

**DOI:** 10.1017/S095026881800273X

**Published:** 2018-10-23

**Authors:** A. C. Teirlinck, B. de Gier, A. Meijer, G. Donker, M. de Lange, C. Koppeschaar, W. van der Hoek, M. E. Kretzschmar, S. A. McDonald

**Affiliations:** 1 Centre for Infectious Disease Control, National Institute for Public Health and the Environment (RIVM), PO Box 1, 3720 BA Bilthoven, Netherlands; 2 Nivel Primary Care Database – Sentinel Practices, Utrecht, Netherlands; 3 De Grote Griepmeting, Science in Action BV, Amsterdam, Netherlands; 4 Julius Center for Health Sciences and Primary Care, University Medical Center Utrecht, Utrecht, The Netherlands

**Keywords:** Evidence synthesis, incidence, influenza (seasonal), statistical modelling

## Abstract

Due to differences in the circulation of influenza viruses, distribution and antigenic drift of A subtypes and B lineages, and susceptibility to infection in the population, the incidence of symptomatic influenza infection can vary widely between seasons and age-groups. Our goal was to estimate the symptomatic infection incidence in the Netherlands for the six seasons 2011/2012 through 2016/2017, using Bayesian evidence synthesis methodology to combine season-specific sentinel surveillance data on influenza-like illness (ILI), virus detections in sampled ILI cases and data on healthcare-seeking behaviour. Estimated age-aggregated incidence was 6.5 per 1000 persons (95% uncertainty interval (UI): 4.7–9.0) for season 2011/2012, 36.7 (95% UI: 31.2–42.8) for 2012/2013, 9.1 (95% UI: 6.3–12.9) for 2013/2014, 41.1 (95% UI: 35.0–47.7) for 2014/2015, 39.4 (95% UI: 33.4–46.1) for 2015/2016 and 27.8 (95% UI: 22.7–33.7) for season 2016/2017. Incidence varied substantially between age-groups (highest for the age-group <5 years: 23 to 47/1000, but relatively low for 65+ years: 2 to 34/1000 over the six seasons). Integration of all relevant data sources within an evidence synthesis framework has allowed the estimation – with appropriately quantified uncertainty – of the incidence of symptomatic influenza virus infection. These estimates provide valuable insight into the variation in influenza epidemics across seasons, by virus subtype and lineage, and between age-groups.

## Background

Characteristics of the seasonal influenza epidemic that annually occurs in the northern hemisphere winter vary greatly between seasons (usually defined as week 40 through week 20 of the following year). Although an epidemic can be expected to occur every winter, the attack rate, timing, affected age groups and therefore the disease burden of the epidemic can vary widely. Of the drivers of variation in transmission intensity between seasons [1], the dominant type (A or B), subtype (of influenza A) or lineage (of influenza B) of the circulating influenza viruses is a key factor. Furthermore, due to genetic drift in influenza viruses, the virulence, antigenic match to vaccine strains and pre-existing immunity for a particular virus subtype/lineage can also vary substantially from one season to the next [2].

At the national level, a frequently applied – although indirect – approach for monitoring the seasonal influenza epidemic and measuring variation in intensity across seasons is through the incidence of influenza-like illness (ILI) from sentinel general practitioner (GP) networks, and/or the incidence of severe acute respiratory infection (SARI) from hospital surveillance [3]. Because of overlapping clinical symptoms with influenza, infections with other respiratory viruses such as rhinovirus and respiratory syncytial virus (RSV) also contribute to the observed ILI and SARI incidence. As with influenza virus, the circulation of other respiratory viruses also differs between seasons, which means that reliance on ILI or SARI data alone does not provide an accurate picture of the influenza epidemic intensity.

Furthermore, for the calculation of population-level influenza burden [4] (used by policy makers to compare the disease burden attributable to various infectious agents), *influenza-associated* ILI or SARI incidence is the recommended indicator, which takes into account the proportion of ILI/SARI cases that are influenza virus-positive [3, 5, 6]. A weakness of influenza-associated ILI/SARI incidence is that it is restricted to medically attended cases only; patients with only mild clinical symptoms and patients who self-medicate tend not to visit a healthcare facility, which results in underestimation of the extent of the influenza burden. To obtain a better estimate of the incidence of symptomatic influenza – not just ILI/SARI – in the community, methods are required that appropriately integrate multiple data sources [7–10].

In the current study, we aim to estimate the incidence of *symptomatic infection with influenza virus* (hereafter, SI) in the Netherlands, stratified by season and by age-group and to compare SI incidence across seasons according to the predominantly circulating influenza virus A subtype and B lineage. These stratified SI incidence estimates are vital for the calculation of disease burden, economic burden (including healthcare costs) and for investigating the effects of preventive measures at the national level [11]. Although asymptomatic influenza cases can play a role in transmission, for purposes of disease burden estimation, asymptomatic cases are irrelevant and so we restricted our analyses to SI only.

## Methods

In a previous study, we had estimated SI incidence for a ‘typical’ influenza season within the Bayesian multi-parameter evidence synthesis (MPES) framework, by aggregating the relevant data over the period 2005–2007 [12]. Here, we extend this statistical modelling approach to produce separate estimates for the individual seasons comprising a six-season period, with 95% uncertainty intervals (UIs, i.e. from the 2.5% to the 97.5% percentile), taking into account potential autocorrelation in ILI incidence rates across seasons.

In the main analysis – and as an improvement over previous work [12] – we restricted analysis to the winter season only (week 40 through week 20 of the following year). Because there is very little influenza virus circulating, very few ILI patients are sampled for virological testing outside these weeks (for a separate summer period analysis, i.e. for week 21 through week 39, see Supplementary Material, Fig. S3). Therefore, data from the period beginning week 40 of 2011 through week 20 of 2017 were used.

### Data sources

#### ILI incidence

The number of patients that consulted their GP with ILI were obtained from the Nivel Primary Care Database sentinel surveillance [13]. Since the start of sentinel ILI surveillance in the Netherlands in 1970, an ILI case has been defined as follows: sudden onset of symptoms, fever ⩾38 °C and at least one of the following symptoms: cough, rhinorrhoea, sore throat, frontal headache, retrosternal pain or myalgia [14]. Weekly ILI incidence was calculated stratified by the age-group (<5, 5–14, 15–44, 45–64, 65+ years), using the GP-enlisted population by age as a denominator. The catchment population for the sentinel practices consists of approximately 0.7% of the Dutch population, and is representative for age, sex, regional distribution and population density [13].

#### Underascertainment factor

Internet-based monitoring of ILI in the general population (Influenzanet [15, 16]) provided data on season- and age-group specific numbers of respondents reporting ILI and the numbers of respondents reporting ILI who contacted their GP. A case definition for self-reported ILI symptoms comparable with that used for the Nivel sentinel surveillance was applied (ILI^hist^ from Ref. [16]).

#### Influenza virus positivity rate

A subset of patients who presented with ILI at a Nivel sentinel GP were sampled by taking a nose swab and throat swab; these were combined into a single tube with virus transport medium for virological diagnostic testing by real-time reverse-transcription polymerase chain reaction (PCR) (RT-PCR) at RIVM [17]. A patient was considered influenza virus positive regardless of co-infection. Influenza virus type A positive specimens were haemagglutinin (H) and neuraminidase (N) subtyped by RT-PCR, and for influenza virus type B positive specimens, the lineage was determined by RT-PCR. The number of positive influenza virus tests and number of tested specimens, per season, age-group and per strain/lineage (A(H1N1)pdm09, A(H3N2), B (Victoria lineage) and B (Yamagata lineage)) were used for the analysis. The analytical sensitivity of virological testing was estimated at below 4000 digital genome copies per ml specimen, based on a 100% correct score in annual External Quality Assessment studies conducted by Quality Control for Molecular Diagnostics (QCMD), Glasgow, Scotland, a requirement for the RIVM laboratory being accredited according to the ISO 15189 norm. PCR is the modern gold standard for laboratory diagnosis of an influenza virus infection, and the clinical sensitivity is considered to be 95–100%, if a specimen has been correctly taken and in the correct time period after onset of symptoms. Virus shedding peaks at around 1–2 days after onset of symptoms, after which – for normally healthy persons – it usually declines to undetectable levels 7 days after onset of symptoms [18]. Therefore, we restricted the laboratory diagnostic data included in this study to those patients diagnosed with ILI from whom a specimen had been collected not more than 7 days post-symptom onset.

### Synthesis of all relevant data sources

We implemented a previously published approach for estimating SI incidence by combining all relevant data with *a priori* knowledge regarding model parameters underlying SI incidence, via Bayesian MPES [12]. MPES is a means for making use of all available information, and is suitable for estimation of epidemiological parameters such as prevalence or incidence for which no direct measurements exist, but which may be indirectly informed by other data sources. Furthermore, Bayesian models can easily account for correlation between parameters if they are estimated jointly. This estimation approach can be viewed as similar to the ‘multiplier method’ or ‘direct method’ often used for estimating epidemiological parameters [19, 20], but with correct propagation of the uncertainty associated with each data source to the final estimate. As an extension of our previous approach, we estimated season-specific SI influenza incidence rates allowing for the non-independence in ILI incidence rates across seasons. Either a positive or negative correlation in ILI rates across time could result from changes in the dominance of circulating influenza virus subtypes/lineages, virulence and antigenic drift, as well as population factors related to exposure during previous seasons.

To investigate between-season variation in SI incidence according to influenza virus A subtype and B lineage, we also specified a model in which data were stratified by subtype (A(H1N1)pdm09 and A(H3N2)) and lineage (B/Victoria, B/Yamagata). We report SI incidence by dominant subtype/lineage, and with respect to the presence of a co-circulating strain(s) (in which co-circulation was defined as two (sub)types that each represented 40–60% of all influenza virus detections). Due to low counts, it was not feasible to stratify by subtype/lineage and age-group concurrently.

### Model specification and inference

The incidence of SI, *N*_SI_, stratified by season and age-group, can be estimated using the ‘multiplier method’, as:

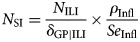

where *N*_ILI_ is the total number of ILI patients from sentinel surveillance, *δ*_GP|ILI_ refers to the proportion of persons reporting ILI who consult their GP (from Influenzanet), *ρ*_Infl_ is the influenza virus positivity rate from virological testing and *Se*_Infl_ is the estimated clinical sensitivity of this test. The above equation represents a ‘user-friendly’ version of the directed acyclic graph (DAG) stipulating the relationships (stochastic and deterministic) between the parameters and data sources (see Supplementary Material, S1 and Fig. S4) that comprise the MPES model.

The main task of the MPES model is to estimate *N*_SI_. Data sources and other required parameters are described in Table 1, and model equations and example OpenBUGS code are provided in Supplementary Material, S3. Sampling from the posterior distribution for each parameter was performed using OpenBUGS [21] (two chains with a burn-in of 50 000 iterations, with a further 40 000 iterations retained). The posterior distribution of the season- and age-group stratified SI parameters were summarised using the median and the 95% UI, which were then converted to incidence rates per 1000 population for ease of comparison between the seasons and across age-groups. The extent to which SI incidence was under- or over-estimated by medically attended ILI alone (i.e. clinical illness but not necessarily confirmed influenza) was indicated through calculation of multiplication factors [22], which were defined separately for each season and age-group.

**Table 1.** Data sources and model parameters, adapted from Ref. [12]

Parameter	Distribution/functional form	Evidence
*c*_*a*,*t*,ILI*|*Pop_	See Supplementary Material, S2	Indirect evidence from observed ILI cases (GP consultations) from sentinel surveillance
*d*_*a*,*t*,ILI_	Beta(0.5,0.5)	Data from the Influenzanet study [15]: age-group specific numbers of self-reported ILI cases, and ILI cases who visited a GP
*c*_*a*,*t*,SI*|*ILI_	Beta(0.5,0.5)	Direct evidence from virological testing done on random sample of ILI cases, age-group specific
*Se*_Infl_	Uniform(0.95,1.0)	Assumed clinical test sensitivity for influenza virus
*N*_*a*,*t*,Pop_	N/A	Population size estimates (Statistics Netherlands)
*N*_*a*,*t*,ILI_	Bin(*N*_*a*,*t*,Pop_, *c*_*a*,*t*,ILI|Pop_)	Observed sentinel surveillance data on ILI cases; binomial likelihood for observed data with detection probability *d*_*a*,*t*,ILI_
*N*_*a*,*t*,SI_	Bin(*N*_*a*,*t*,ILI_, *c*_*a*,*t*,SI*|*ILI_)	All model assumptions and data

Our model was developed under the assumption that ILI incidence rates over the different seasons should not be considered as independent data. Considering ILI incidence rates over a long (21-seasons) analysis period, there is a small positive lag-1 correlation in this parameter apparent for all age-groups (Supplementary Material, S2 and Fig. S1). We reasoned that for identical parameters estimated across multiple seasons, one should allow for possible non-independence of these parameters across time. The origin of a positive temporal dependence in ILI incidence rates between season *i* and season *i* − 1 is multifactorial; for instance, genetic drift of the virus, subtype and lineage dominance, waning immunity rate [11], and relatively low transmission intensity. Such positive temporal correlations would be offset by a potentially negative correlation due to natural immunity at the population level when an antigenically similar (dominant) subtype or lineage virus is widely circulating in both season *i* and season *i* − 1. Thus, we defined the priors for the ILI rates to allow for potential autocorrelation, by specifying an autoregressive process of order 1, AR(1).

## Results

### Season-specific SI incidence rates

The age-aggregated incidence rates of SI with influenza per 1000 persons were 6.5 (95% UI: 4.7–9.0) for season 2011/2012, 36.7 (95% UI: 31.2–42.8) for 2012/2013, 9.1 (95% UI: 6.3–12.9) for 2013/2014, 41.1 (95% UI: 35.0–47.7) for 2014/2015, 39.4 (95% UI: 33.4–46.1) for 2015/2016 and 27.8 (95% UI: 22.7–33.7) for season 2016/2017; see Supplementary Material, Table S1. In the total Dutch population, these rates correspond to 109 thousand, 616 thousand, 153 thousand, 694 thousand, 670 thousand and 475 thousand SI cases in seasons 2011/2012 through 2016/2017, respectively.

#### Age-specific SI incidence rates

**Fig. 1.**
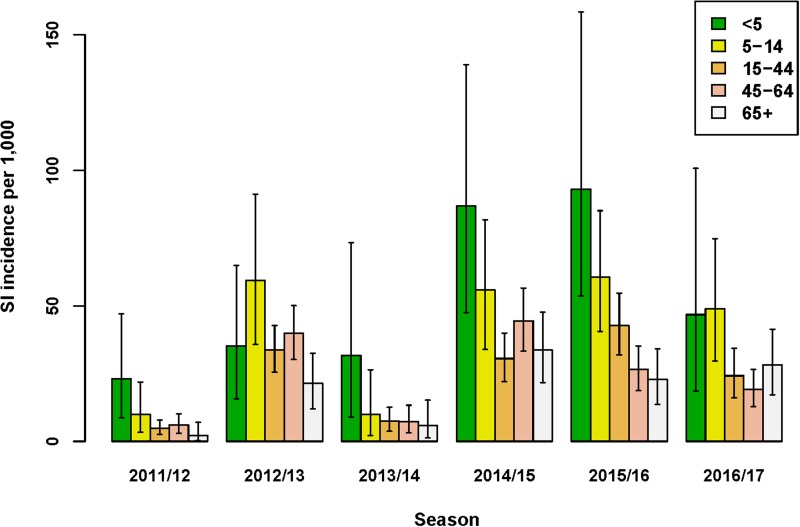
Estimated incidence of symptomatic infection (SI) with influenza virus per 1000 population for seasons 2011/12 through 2016/17, winter period only. Lines indicate 95% UIs.

The incidence of SI was highest for the age-group <5 years in four out of six seasons (Fig. 1 and Supplementary Material, Table S1). Over all seasons, the median difference in SI incidence between the youngest (<5 years) and older (5–14 years) children was 13.2/1000 (95% UI: −5.0 to 34.4). Of the estimated total number of SI cases, the proportion among the <5 years age-group ranged between 5% and 19%, and the proportion among 65+ years also ranged between 5% and 19% across seasons. Among all age groups from 5 years and older, SI incidence varied per season, but between-age group differences were relatively small. Over all seasons, the lowest SI incidence among the 5–14 through 65+ years age-groups was observed for the 65+ age-group; the median difference in SI incidence between this age-group and the next youngest (45–64 years) age-group was 4.3/1000 (95% UI: −0.9 to 9.3).

The age-group pattern differed if influenza virus positivity and underascertainment were excluded from the MPES; considering only those ILI patients who consult their GP, the highest and second highest ILI incidence rates were observed for the <5 and 65+ years age-groups, respectively (Supplementary Material, Table S2). Furthermore, reliance on medically attended ILI rates alone would either under- or over-estimate SI incidence (Supplementary Material, Fig. S4). For instance, in the 2016/17 season ILI underestimated SI incidence in all age-groups under 65+ years, yielding multiplication factors of 1.4–2.9, but in season 2011/12 among persons aged 65+ years SI incidence was substantially *over*-estimated by medically attended ILI (MF = 0.16), mainly due to the low proportion of influenza positives among swabbed ILI patients in this stratum. In contrast to SI incidence, ILI incidence was always underestimated by medically attended ILI (Fig. S4), because underascertainment factors were always positive.

#### Influenza A subtypes and B lineages among swabbed ILI patients

**Fig. 2.**
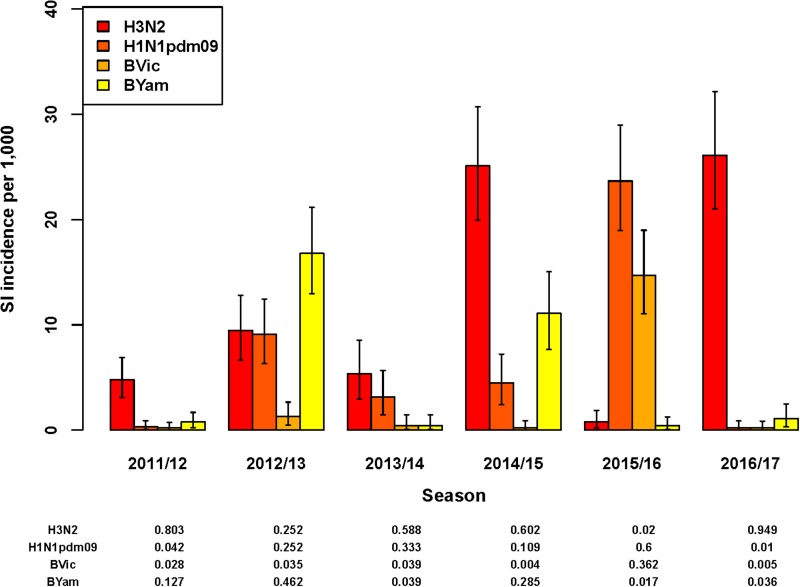
Estimated incidence of symptomatic infection (SI) with influenza virus per 1000 population for seasons 2011/12 through 2016/17 by A subtype and B lineage, winter period only. Lines indicate 95% UIs. Table below figure shows proportion of all influenza virus detections per season.

In five of the six seasons assessed, an influenza virus A subtype was dominant in terms of the highest SI incidence among all four subtypes/lineages (Fig. 2). For season 2012/2013 only, influenza virus B (Yamagata lineage) had the highest SI incidence of all subtypes/lineages. However, as influenza A and B were co-dominant in this season only (51% and 49% of all detections for influenza A and B, respectively), the SI incidence of the two influenza A virus subtypes combined (18.6/1000; 95% UI: 14.5–23.5) was highly similar to that of the two influenza B virus lineages combined (18.1/1000; 95% UI: 14.2–22.7). In all other seasons, both influenza virus types A and B were detected, but there was a clear difference in proportions between seasons (Fig. 2) and co-dominance was not observed. Influenza virus type A generally preceded influenza virus type B circulation (data not shown) during the course of the season. Seasons 2014/2015 and 2016/2017 had very similar SI incidence for influenza virus A(H3N2), but in the 2014/2015 season influenza virus A(H1N1)pdm09 and influenza virus B (Yamagata lineage) additionally circulated, resulting in a much higher total SI incidence.

Total SI incidence tended to be highest in those seasons in which both an A subtype and a B lineage were dominant contributors. Aggregating over all six seasons, A(H3N2) resulted in more symptomatic infections than any of the other subtypes/lineages (SI incidence of 12.0/1000; 95% UI: 10.6–13.6); the next highest was A(H1N1)pdm09 (SI incidence of 6.9/1000; 95% UI: 5.8–8.1).

## Discussion

We estimated the incidence of symptomatic infection with influenza virus in the Netherlands, stratified by season and by age-group and compared SI incidence across seasons according to virus A subtype or B lineage (Fig. 2). This modelling study has generated estimates of season-specific SI incidence in the Netherlands across six seasons, 2011/2012 through 2016/2017. The MPES approach enables useful comparisons of the variation in intensity (as SI) across virus A subtypes/B lineages and between age-groups, as uncertainty in estimated incidence is computed. Age-group differences in virological positivity rates and healthcare-seeking behaviour among patients with ILI symptoms were integrated with ILI rates from sentinel GP surveillance.

Accounting for underascertainment of ILI represents an improvement over influenza intensity indicators that are restricted to medically attended ILI cases; measurement of GP-attended ILI only yields divergent age-group patterns compared with SI incidence (Supplementary Material, Table S2) and thus would invite a different interpretation. SI incidence estimates are also important for estimating the subtype/lineage- and age-stratified morbidity burden of influenza, and they may serve as inputs for cost-effectiveness studies that assess national influenza prevention strategies such as vaccination.

Interestingly, in the 2015/2016 season, in which influenza virus subtype A(H1N1)pdm09 was dominant, estimated SI incidence was lowest for the 65+ years age-group, which is consistent with the observation that people born before 1957 have better pre-existing immunity to influenza virus subtype A(H1N1) that was dominantly circulating from 1918 until 1957 [23], compared with people born since then. Although it is known that the elderly are disproportionally over-represented among all influenza-associated hospitalisations and deaths [19, 24–27], in the case of influenza with a relatively mild clinical course (i.e. ILI), this age-disadvantage is not observed. In most seasons, SI incidence was lower among the 65+ years age-group compared with the younger age groups (Fig. 1), consistent with what has been reported by others [5, 6, 20]; this is possibly related to the fact that this age-group is targeted for vaccination, has stronger natural immunity [28] and/or has lower rates of contact [29] with infectious persons.

The well-established influenza surveillance in Europe is primarily based upon medically attended ILI incidence, to monitor the characteristics – timing and intensity – of an influenza season. In the Netherlands, the long-standing sentinel GP surveillance system has provided extremely valuable information on ILI since 1970 [13]. However, for better estimation of influenza incidence and influenza-associated disease burden, ILI alone does not suffice: an unknown number of influenza patients may have an acute respiratory infection but do not fit the ILI case definition (low sensitivity), and a substantial proportion of ILI episodes will not have been caused by influenza virus infection (low specificity) [17]. As may be anticipated from surveillance in a routine primary care setting, ILI diagnoses are a mix of applying an agreed case definition and clinical experience. In addition, we have shown that reliance on medically attended ILI alone (unadjusted for virological confirmation) as a quantitative indicator of influenza intensity may either under- or over-estimate SI incidence.

Strengths of our study include the use of all available, relevant data sources for the estimation of SI incidence and the extension of our previous approach [12] to provide season-specific estimates [9, 10], stratified additionally by age-group or by influenza virus A subtype/B lineage. Several limitations of the data sources must be also considered when interpreting the model estimates. First, the likelihood of consulting a GP given ILI symptoms (as captured by Influenzanet data) may be affected by bias, if health-seeking behaviour differs between the internet users and non-internet users. Also, children and elderly persons are known to be under-represented by this internet-based survey, but this is only an issue if age differences in health-seeking behaviour are confounded with frequency of internet use. Given that that the underascertainment parameter is an important determinant of estimated SI incidence, further investigation of this issue is needed. Second, we applied Influenzanet data as if these data were free from sampling bias or misclassification errors, as we had no external information to estimate these. This means that the degree of uncertainty associated with the parameter *δ*_GP|ILI_ is narrower than in reality. Note that this also applies to other observed data, for which no information on measurement error was available. Third, the precision of influenza virus positivity rates was limited by the relatively few patients swabbed per GP practice per week. However, restriction of analyses to the winter period (week 40 through week 20) ensured that weeks with extremely sparse data did not contribute to seasonal totals. Additionally, it was assumed that the swabbed ILI patient population is an unbiased representation of all ILI patients who consult the GP, but sampling of ILI patients conducted randomly.

Finally, GP sentinel surveillance only delivered data for community dwelling elderly persons. The elderly living in nursing homes were excluded, as they would normally not consult physicians outside the facility. This may have affected the estimated SI incidence for the 65+ years age-group. However, in 2015 approximately 117 000 persons lived in nursing or elderly homes in the Netherlands [30]; this figure represents only about 10% of the total 65+ years population. Even if ILI incidence was two to three times greater in nursing homes than among community dwelling elderly (as suggested by sparse data from nursing home surveillance [17]), this would not significantly impact our estimates for this age-group.

In conclusion, integrating all relevant data sources – season-specific ILI surveillance, virological testing and data on healthcare-seeking behaviour – has allowed estimation of SI incidence. Compared with the ‘direct method’, the evidence synthesis framework adds value in terms of flexibility (allows incorporation of new data sources that either directly or indirectly inform individual parameters; as well, observed temporal trends can be modelled), and appropriately quantified uncertainty. These estimates provide valuable insight into the variation in influenza epidemic intensity across seasons, by influenza virus A subtype and B lineage and between age groups.

## Supplementary material

For supplementary material accompanying this paper visit https://doi.org/10.1017/S095026881800273X. click here to view supplementary material
