# Supplementary material for: The incidence of symptomatic infection with influenza virus in the Netherlands 2011/2012 through 2016/2017, estimated using Bayesian evidence synthesis
Source: Epidemiol Infect. 2018 Oct 23;147:e30. doi: 10.1017/S095026881800273X (PMC6518592; doi:10.1017/S095026881800273X)

Epidemiology and Infection

*Title:*

The incidence of symptomatic infection with influenza virus in the Netherlands 2011/2012 through 2016/2017, estimated using Bayesian evidence synthesis

*Authors:*

Anne C. Teirlinck, Brechje de Gier, Adam Meijer, Gé Donker, Marit de Lange, Carl Koppeschaar, Wim van der Hoek, Mirjam E. Kretzschmar, Scott A. McDonald

**Supplementary Material**

**Table S1:** Estimated incidence of symptomatic infection with influenza virus (SI), for seasons 2011/12 through 2016/17 (winter period only) and aggregated over all six seasons.

| **Age group** | **SI per 1,000** (median with 95% uncertainty interval) | | | | | |  |
| --- | --- | --- | --- | --- | --- | --- | --- |
|  | **2011/12** | **2012/13** | **2013/14** | **2014/15** | **2015/16** | **2016/17** | **(All)** |
| <5 | 23.0 | 35.2 | 31.7 | 86.9 | 93.0 | 46.8 | 53.8 |
|  | 8.7-47.1 | 15.6-64.9 | 8.9-73.3 | 47.6-139.0 | 53.7-158.5 | 18.5-100.7 | 39.8-74.1 |
| 5-14 | 10.0 | 59.4 | 10.0 | 55.9 | 60.6 | 48.9 | 41.2 |
|  | 3.3-21.9 | 35.7-91.3 | 2.2-26.4 | 33.9-81.7 | 40.6-85.1 | 29.6-74.7 | 32.5-51.9 |
| 15-44 | 4.8 | 33.8 | 7.5 | 30.6 | 42.7 | 24.2 | 24.0 |
|  | 2.5-7.8 | 25.6-42.8 | 3.7-12.6 | 22.0-39.9 | 32.0-54.7 | 16.1-34.3 | 20.8-27.4 |
| 45-64 | 6.1 | 39.8 | 7.4 | 44.5 | 26.5 | 19.2 | 24.0 |
|  | 3.0-10.1 | 30.3-50.1 | 3.1-13.3 | 33.3-56.6 | 18.7-35.2 | 12.9-26.6 | 20.9-27.4 |
| 65+ | 2.2 | 21.4 | 5.9 | 33.6 | 22.8 | 28.2 | 19.7 |
|  | 0.3-7.0 | 12.0-32.5 | 1.3-15.2 | 21.7-47.6 | 13.6-34.1 | 17.2-41.3 | 15.9-24.1 |
| All | 6.5 | 36.7 | 9.1 | 41.1 | 39.4 | 27.8 |  |
| ages | 4.7-9.0 | 31.2-42.8 | 6.3-12.9 | 35.0-47.7 | 33.4-46.1 | 22.7-33.7 |  |

**Table S2.** Posterior incidence of GP-attended influenza-like illness (ILI) per 1,000 population (median estimate with 95% uncertainty interval), for seasons 2011/12 through 2016/17, winter period only.

| **Age group** | **2011/12** | **2012/13** | **2013/14** | **2014/15** | **2015/16** | **2016/17** |
| --- | --- | --- | --- | --- | --- | --- |
| <5 | 39.3 | 60.0 | 49.4 | 76.6 | 67.7 | 67.2 |
|  | 34.5-44.7 | 53.9-66.5 | 43.8-55.6 | 70.1-84.2 | 61.6-74.1 | 61.1-73.8 |
| 5-14 | 9.1 | 18.8 | 10.3 | 20.9 | 21.1 | 15.9 |
|  | 7.5-10.8 | 16.5-21.3 | 8.6-12.2 | 18.6-23.5 | 18.8-23.5 | 13.9-18.1 |
| 15-44 | 9.1 | 17.8 | 9.7 | 19.0 | 15.0 | 13.3 |
|  | 8.2-10.1 | 16.5-19.1 | 8.8-10.8 | 17.7-20.4 | 13.9-16.2 | 12.3-14.4 |
| 45-64 | 10.2 | 21.6 | 12.1 | 25.5 | 19.3 | 17.6 |
|  | 9.1-11.4 | 20.0-23.3 | 10.9-13.4 | 23.8-27.4 | 17.8-20.9 | 16.2-19.1 |
| 65+ | 13.4 | 22.2 | 16.3 | 31.3 | 24.0 | 27.9 |
|  | 11.8-15.2 | 20.2-24.4 | 14.6-18.2 | 29.0-33.8 | 21.9-26.1 | 25.7-30.2 |

**S1 Evidence synthesis model equations**

The primary goal of the model is to compute the posterior distribution of the parameter *N*_SI_, the number of cases of symptomatic infection (SI) with influenza (Fig. S3). Model parameters relate the numbers in each subpopulation (i.e., ascertained ILI cases, all ILI cases, all ILI cases testing influenza positive, and all SI cases) through stochastic relationships, as conditional probabilities. We assume that the actual numbers in the subpopulations *N*_ILI_ and *N*_SI_ are binomially distributed.

The probability of ILI occurring in the population, *c*_a,ILI|Pop_, is informed by data on the annual number of ILI patients provided by the sentinel GP surveillance system and the estimated under-ascertainment. The number of sampled ILI cases, *y*_a,GP_, was assumed binomially distributed, with priors on the detection probability *d*_a,ILI_ specified using age-group specific ascertainment estimated from the internet-based monitoring (Influenzanet) study (Friesema et al. 2009). Ascertainment was defined based on the age-group specific number of (self-reported) ILI cases in the Influenzanet study, *n*_a,GIS_, and the number of ILI cases from the same study who reported visiting a GP, *y*_a,GIS_.

A vague Jeffries prior, *Beta*(0.5,0.5), was specified for the ascertainment parameter *d*_a,ILI_. Data on the number of ILI patients reported by the sentinel GP network, *y*_a,GP_, and the size of the sentinel population, *n*_a,GP_, were used (together with estimated ascertainment to account for those ILI cases not consulting a GP) to inform the conditional probability *c*_a,ILI|Pop_. The model is specified as the following:

*N*_a,t,ILI_ ~ Binomial(*N*_a,t,Pop_, *c*_a,t,ILI|Pop_)

*N*_a,t,SI_ ~ Binomial(*N*_a_,_t,ILI_, *c*_a,t,SI|ILI_)

*y*_a,t,GIS_ ~ Binomial(*n*_a,t,GIS_, *d*_a,t,ILI_)

*y*_a,t,GP_ ~ Binomial(*n*_a,t,GP_, *d*_a,t,ILI_ × *c*_a,t,ILI|Pop_)

where:

*c*_a,t,ILI|Pop_ Probability of ILI in the population

*c*_a,t,SI|ILI_ Probability of symptomatic influenza infection given ILI

*d*_a,t,ILI_ Proportion of true ILI cases that are reported

From the above equations (and Fig. S3) it can be seen that the conditional probability of being infected with influenza given ILI is informed by data from virological testing of samples provided by the sentinel GPs, for which the number of positive cases by age-group *a* and per respiratory year *t*, *y*_a,t,VIR_, and the denominator (ILI samples tested), *n*_a,t,VIR_, were available. This probability also depends on the sensitivity of the test, π, which was assigned a uniform prior ranging from 95% to 100%. The test sensitivity was incorporated into the binomial likelihood for the observed number of positive samples *y*_a,t,VIR_. The uninformative prior, *Beta*(0.5,0.5), was specified for the conditional probabilities *c*_a,SI|ILI_.

π ~ Uniform(0.95,1)

*y*_a,t,VIR_ ~ Binomial(*n*_a,t,VIR_, π × *c*_a,SI|ILI_)

To model potential autocorrelation, we specify the prior distribution for [the logit transform of] the proportion *c_t,_*_ILI|Pop_ – separately for each combination of age-group and period of season – as the below (ϕ is the parameter controlling the degree to which the random walk approaches the mean):

logit[ *c_a,t,_*_ILI|Pop_) ] ~ *N*(μ + ϕ* logit[*c_a,t-1,_*_ILI|Pop_ ], σ^2^)

Vague priors were specified for μ, ϕ, and τ (=1/σ^2^).

μ ~ Normal(0,1/0.01)

ϕ ~ Normal(0,1)

τ ~ Gamma(0.001,0.001)

**S2 Sensitivity analysis**

We provide this extra analysis for the interested reader.

***Rationale***

It may be incorrect to consider ILI incidence over the different seasons as *independent* data. Fig. S1 shows the autocorrelation functions for ILI incidence rates for five age-groups across the 21 seasons 1991/92 to 2011/12 (Donker, 2012).

Statistical modelling involving temporally-correlated ILI rates should not necessarily discount this dependence across time. Bayesian models can easily account for correlation between parameters if they are estimated jointly. Thus, for the main analysis, we modelled the priors for the parameters *P*(ILI*_t_*|Pop*_t_*) as a variation of a random-walk: an autoregressive model of order 1, AR(1). The expected value of this parameter at time *t* is written as follows (for a given age-group and period of season):

E[ *P*(ILI*_t_*|Pop*_t_*) ] = e*_t-1_* + ϕ *P*(ILI*_t-1_*|Pop*_t-1_*)

To implement this in WinBUGS/OpenBUGS/JAGS, see **S3 Example BUGS code**.

In sensitivity analysis, we explored two other model variants.

1. Independence. In this variant, there is no structure over time; ILI and also symptomatic infection (SI) incidence is estimated separately and independently for each season.

2. Random-walk. The expected value of *P*(ILI*_t_*|Pop*_t_*) at time *t* is written as follows:

E[ *P*(ILI*_t_*|Pop*_t_*) ] = e*_t-1_* + *P*(ILI*_t-1_*|Pop*_t-1_*)

which is implemented in BUGS as:

logit[ *P*(ILI*_t_*|Pop*_t_*) ] ~ *N*(logit[ *P*(ILI*_t-1_*|Pop*_t-1_*) ], σ^2^)

***Results***

Fig. S2 compares the posterior estimates for the incidence of SI with influenza per 1,000 population between model variants, for all seasons and all age-groups. Table S3 shows the posteriors for the number of symptomatic infected cases for a sample season (2014/2015), as estimated by each model variant. Fig. S3 provides the posteriors distributions for the ϕ parameter for the AR(1) model variant. Posterior estimates were largely similar across variants, except that more variation was apparent for the 2014/15 season. 95% uncertainty intervals tended to be wider for the independence model variant.

**Table S3.** Posteriors probabilities of SI incidence for example season 2014/15 by age-group, for all model variants. Values shown are median (95% uncertainty interval).

| Age-group | Main: AR(1) | Independence | Random-walk |
| --- | --- | --- | --- |
| <5 yrs | 77170 (42260-123500) | 76560 (40740-76560) | 74340 (38840-123500) |
| 5-14 yrs | 108300 (65720-158500) | 113100 (62980-113100) | 107000 (60140-166500) |
| 15-44 yrs | 192900 (139100-252000) | 194100 (138500-194100) | 192500 (138900-252300) |
| 45-64 yrs | 211500 (158200-268900) | 215300 (158000-215300) | 212800 (159100-269900) |
| 65+ yrs | 101200 (65320-143300) | 108500 (69180-108500) | 103800 (66190-147000) |

***Model comparison***

We calculated the Deviance Information Criterion (DIC) for each model variant, as a means to compare model fit while trading off complexity (Spiegelhalter et al., 2002); the model with the smallest DIC is the model that makes the best short-term predictions. DIC is the sum of the posterior expected deviance (Dbar), which measures the lack of fit, and the effective number of parameters (*p_D_*). We assume a DIC difference of >10 would rule out the higher-DIC model of the two models being compared.

DIC values for each variant are shown in Table S4. According to this statistic the main model and random-walk variants had similar fits, but the difference between them was negligible. The slightly better fit of the AR(1) (indicated by Dbar) was traded-off by the greater number of effective parameters (pD) compared with the RW variant. The the independence variant had the best lowest DIC and so the best fit to the data. As the difference in DIC between the independence variant and the other two variants was >10 for these data, on this basis one could choose the independence variant. However, given the very small difference in DIC, we selected the AR(1) variant as being consistent with the non-independence in the data – the observed autocorrelation in ILI rates across a longer time-period.

**Table S4.** Comparison of model variants using DIC. Note that DIC = Dbar plus pD

| Statistic | Main: AR(1) | Independence | Random-walk |
| --- | --- | --- | --- |
| DIC | 1150.0 | 1133.0 | 1155.0 |
| *D*bar | 1013.0 | 1010.0 | 1018.0 |
| *p*_D_ | 137.7 | 122.5 | 137.5 |

**S3 Example BUGS code**

model {

## Prior specification

for(a in 1:NG) { # Loop over 5 age groups

# Set vague priors for first season

logitprILIgivenPop[a,1,1] ~ dnorm(0,0.0001)

logitprILIgivenPop[a,1,2] ~ dnorm(0,0.0001)

for(i in 1:NS) { # Loop over each of NS seasons

for(s in 1:2) { # Loop over each period within season

# Pr{ILI | Pop} - from ILI data

logit(prILIgivenPop[a,i,s]) <- logitprILIgivenPop[a,i,s]

# Pr{SI | ILI} - flat prior before data collection

prSIgivenILI[a,i,s] ~ dbeta(0.5,0.5)

Ascert.ILI[a,i,s] ~ dbeta(0.5,0.5)

}

}

for(s in 1:2) {

predlogitpr[a,1,s] <- logitprILIgivenPop[a,1,s]

mu[a,s] ~ dnorm(0,0.01)

phi[a,s] ~ dnorm(0,1)

}

for(i in 2:NS) {

for(s in 1:2) { # Loop over each period within season

predlogitpr[a,i,s] <- mu[a,s] + phi[a,s] * logitprILIgivenPop[a,i-1,s]

logitprILIgivenPop[a,i,s] ~ dnorm(predlogitpr[a,i,s],tau[a,s])

}

}

for(s in 1:2) { # Set vague prior on precision parameter

tau[a,s] ~ dgamma(0.001,0.001)

}

}

Sens.Virol ~ dunif(Sens.Virol.lo,Sens.Virol.hi)

## Posterior specification

for(a in 1:NG) {

for(i in 1:NS) {

for(s in 1:2) {

# Incorporate observed ILI rates & ascertainment data

y.Obs.Prop[a,i,s] ~ dbin(Rate.ILI[a,i,s], n.Obs.Prop[a,i,s])

Rate.ILI[a,i,s] <- (Ascert.ILI[a,i,s] * prILIgivenPop[a,i,s])

x.GPgivenILI.Prop[a,i,s] ~ dbin(Ascert.ILI[a,i,s], n.GPgivenILI.Prop[a,i,s])

# Incorporate observed virological testing data and sensitivity of test

pos[a,i,s] <- (Sens.Virol * prSIgivenILI[a,i,s])

y.Obs.Pos[a,i,s] ~ dbin(pos[a,i,s],n.Obs.Pos[a,i,s])

SAR[a,i,s] <- (prSIgivenILI[a,i,s] * prILIgivenPop[a,i,s])

MF[a,i,s] <- (SI[a,i,s] / (Rate.ILI[a,i,s] * Ntot[a,i,s])

ILI[a,i,s] ~ dbin(prILIgivenPop[a,i,s],Ntot[a,i,s])

SI[a,i,s] ~ dbin(prSIgivenILI[a,i,s],ILI[a,i,s])

}

}

}

## Aggregate over age, stored in index (NG+1)

for(i in 1:NS) {

for(s in 1:2) {

for(a in (NG+1):(NG+1)) {

ILI[a,i,s] <- sum(ILI[1:NG,i,s])

SI[a,i,s] <- sum(SI[1:NG,i,s])

prILIgivenPop[a,i,s] <- ILI[a,i,s] / Ntot[a,i,s]

prSIgivenILI[a,i,s] <- SI[a,i,s] / ILI[a,i,s]

SAR[a,i,s] <- (SI[a,i,s]/ Ntot[a,i,s])

}

}

}

## Aggregate over season, winter period only

for(a in 1:NG) {

SI.agg[a] <- sum(SI[a,1:NS,1])/sum(Ntot[a,1:NS,1])*1000

}

diff.SI.ag1.ag2 <- (SI.agg[1] - SI.agg[2]) # <5 compared with 5-14 yrs

diff.SI.ag5.ag4 <- SI.agg[5] - SI.agg[4] # 65+ compared with 45-64 yrs

}

data {

NG = num_age,

NS = num_season,

Ntot = Pop, # array[age,season,period]

Sens.Virol.lo = SensTestInfl_lo,

Sens.Virol.hi = SensTestInfl_hi,

n.GPgivenILI.Prop = N_reportedILI_GIS, # array[age,season,period]

x.GPgivenILI.Prop = n_GPgivenILI, # array[age,season,period]

y.Obs.Prop = Cases_ILI, # array[age,season,period]

n.Obs.Prop = Popul_ILI, # array[age,season,period]

y.Obs.Pos = PosInfl, # array[age,season,period]

n.Obs.Pos = VirolTest # array[age,season,period]

}

**References**

Donker GA. *Continuous Morbidity Registration at Dutch Sentinel General Practice Network 2011*. Utrecht: NIVEL, Netherlands Institute for Health Services Research; 2012.

Friesema IH, Koppeschaar CE, Donker GA, Dijkstra F, van Noort SP, Smallenburg R, et al.Internet-based monitoring of influenza-like illness in the general population: experience of five influenza seasons in the Netherlands. *Vaccine*, 2009. 27(45): p. 6353-7.

Spiegelhalter DJ, Best NG, Carlin BP, Van Der Linde A. Bayesian measures of model complexity and fit. *J R Statist Soc B* 2002;64(4):583-639.

**Fig. S1**. Autocorrelation plots for ILI rate from the GP sentinel network data covering 21 seasons (1991/92 to 2011/12). The x-axis indicates lag, in seasons.


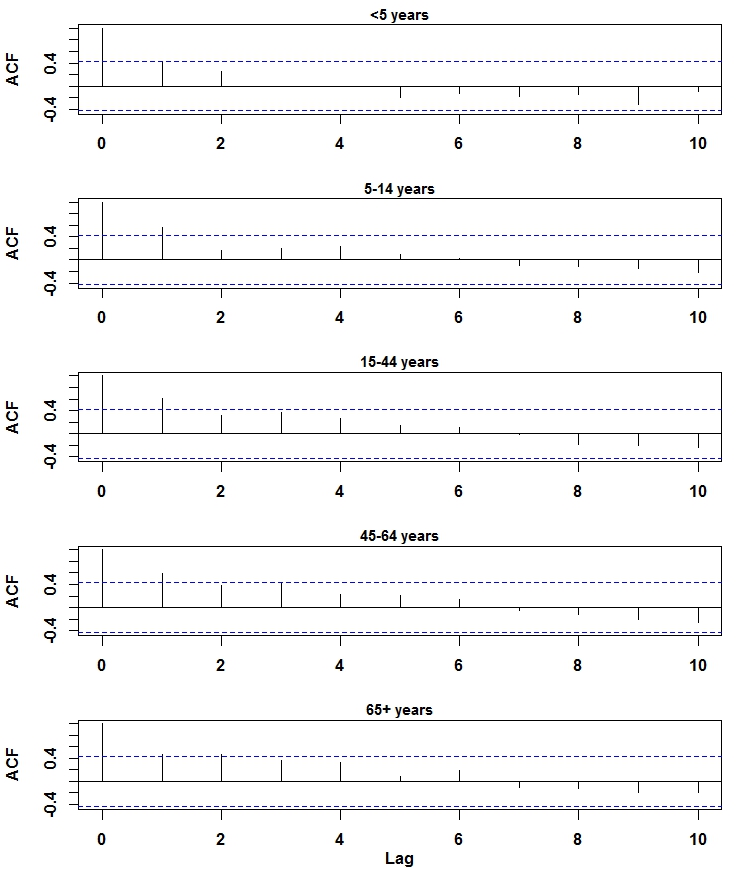


**Fig. S2**. Comparison of the estimated incidence of symptomatic infection with influenza virus (SI) per 1,000 population (winter period only) between three model variants.


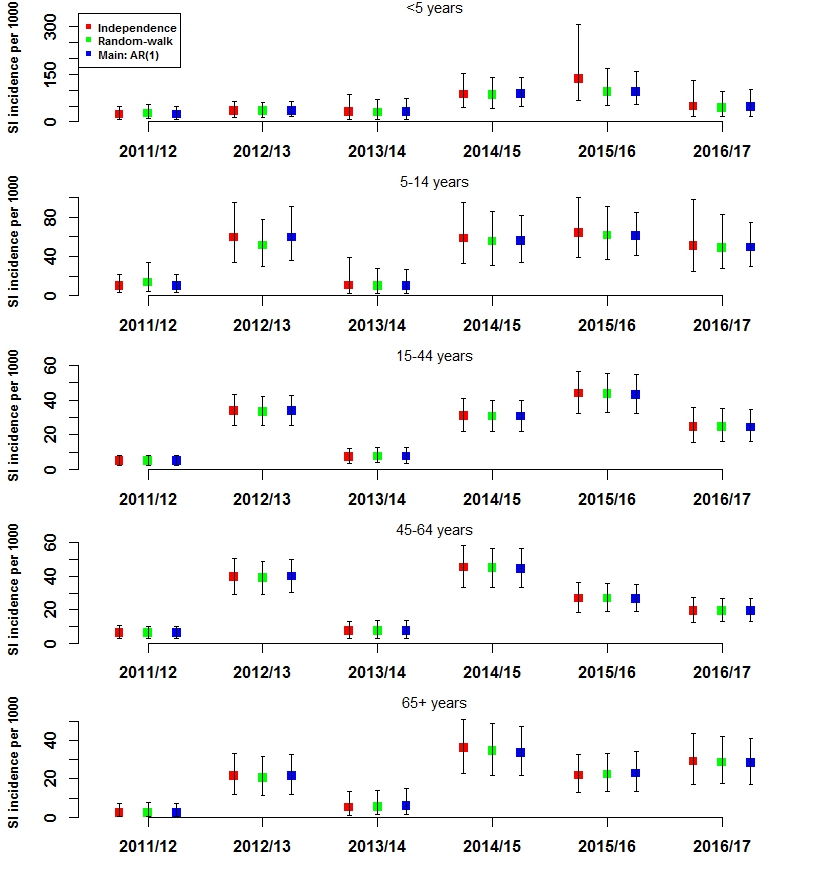


**Fig. S3**. Posterior distributions of the ϕ parameter for the AR(1) (main) model variant, for each age-group, winter period only.

**
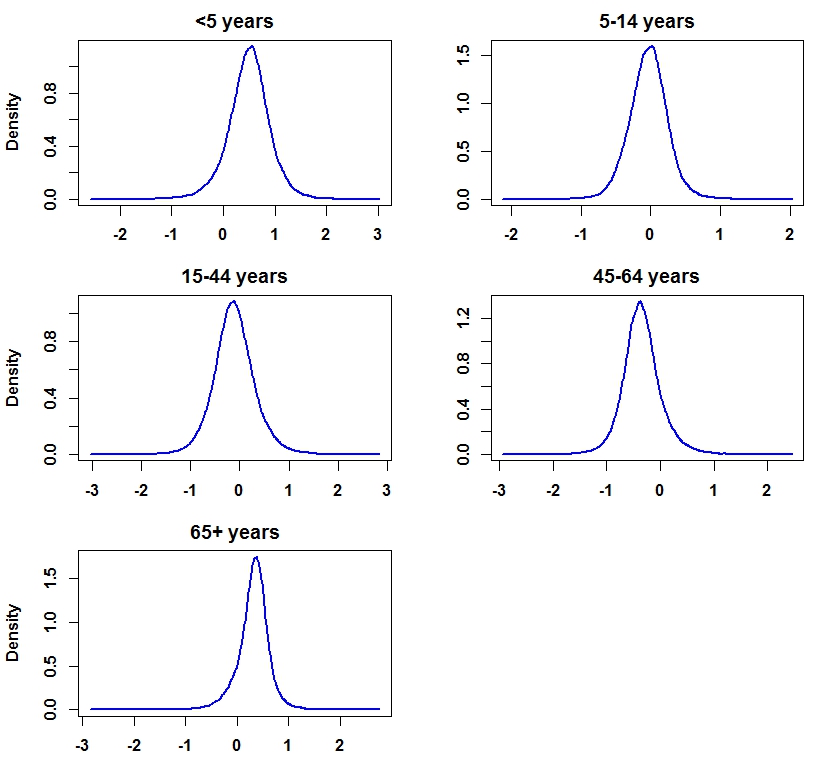
**

**Fig. S4.** Directed acyclic graph of evidence synthesis model showing the relationship between model parameters and observed data; for clarity, only one age-group and one period of the fully stratified model is shown. Stochastic and functional relationships are indicated by solid and dashed lines, respectively. Circles indicate model parameters; double circles indicate parameters for which priors are specified. *d* refers to detection probability (under-ascertainment of ILI cases).


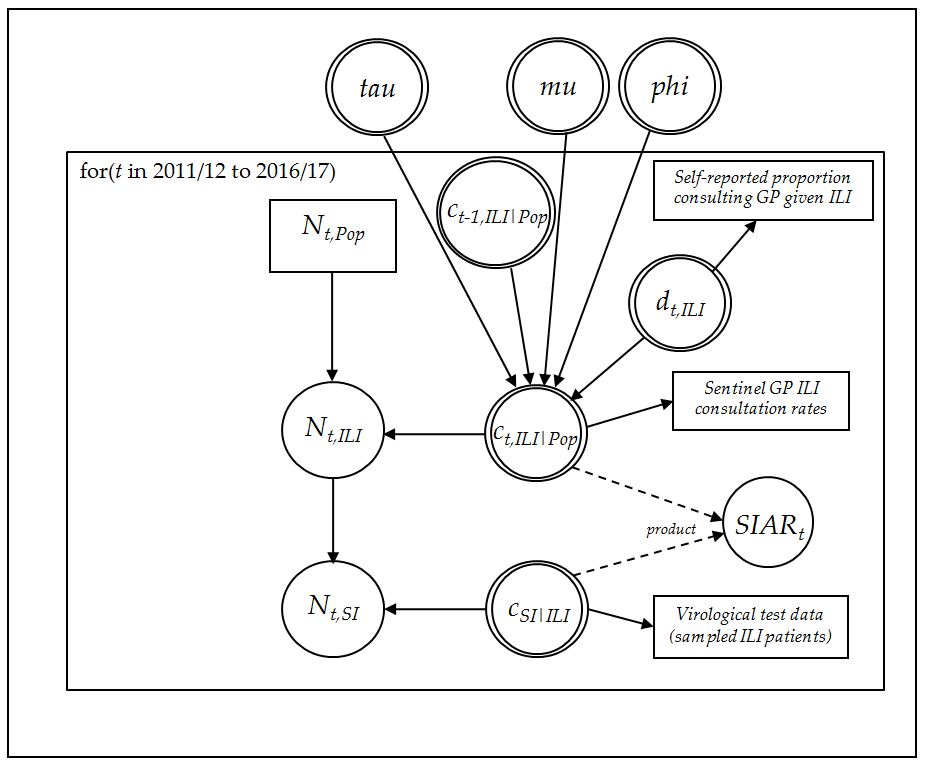


**Fig. S5**. Estimated incidence of symptomatic infection with influenza virus (SI) per 1,000 population; panels show the winter and summer periods separately (NB. data were not yet available for the summer period of season 2016/17). Lines represent 95% uncertainty intervals. Note difference in y-axis scale.

**
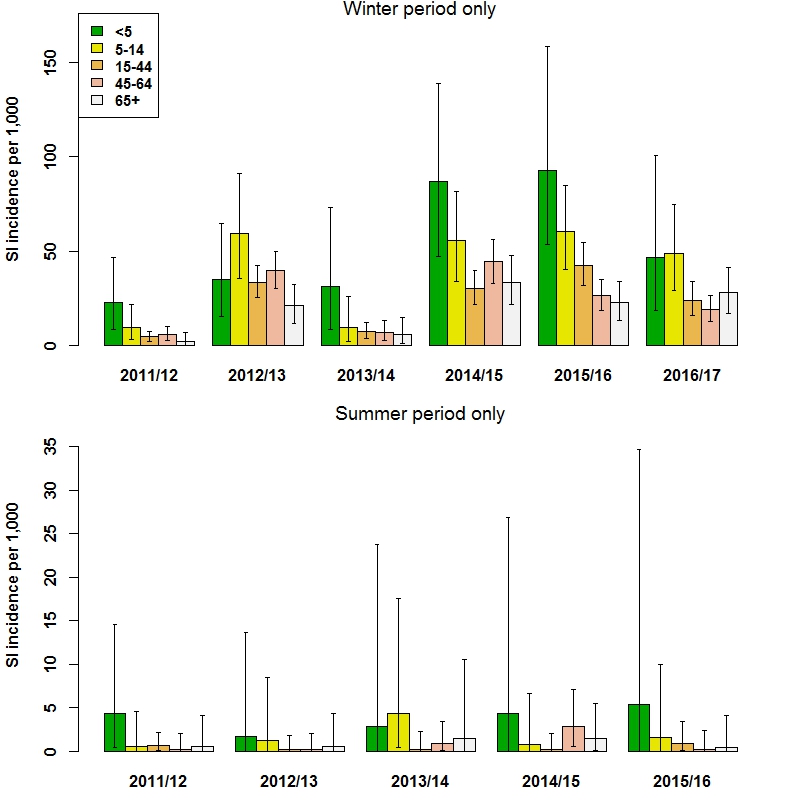
**

**Fig. S6**. Multiplication factors indicating degree of under-estimation (MF >1) or over-estimation (MF<1) of symptomatic influenza infection incidence by medically-attended ILI incidence, by age-group and season (winter period only) (upper panel). Multiplication factors indicating degree of under-ascertainment in ILI incidence by GP-attended ILI incidence (lower panel). Lines represent 95% uncertainty intervals.


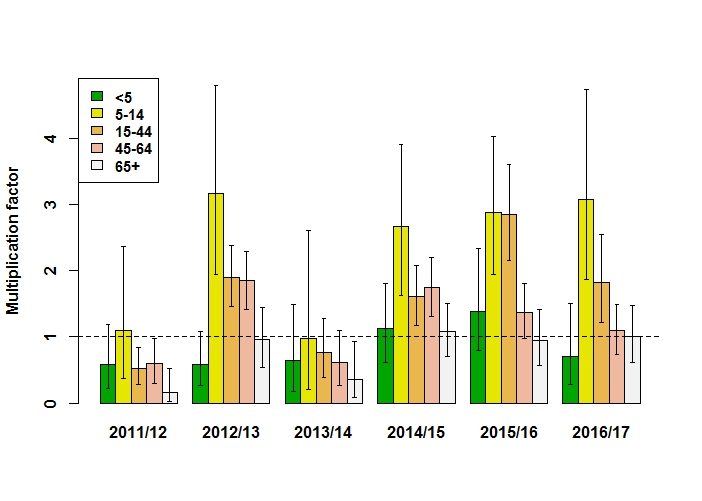


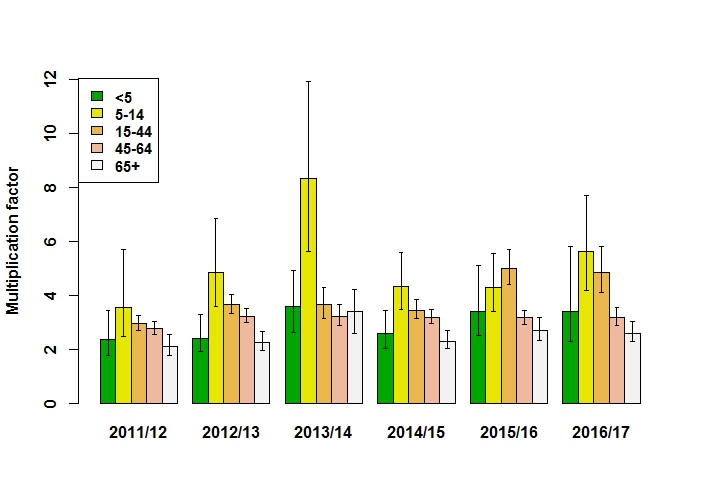

Supplement: Supplementary file 1 [file S095026881800273Xsup001.docx]
